# Phylogenomic instructed target analysis reveals ELAV complex binding to multiple optimally spaced U-rich motifs

**DOI:** 10.1093/nar/gkae826

**Published:** 2024-09-25

**Authors:** David W J McQuarrie, Matthias Soller

**Affiliations:** School of Biosciences, College of Life and Environmental Sciences, University of Birmingham, Edgbaston, Birmingham B15 2TT, UK; Birmingham Centre for Genome Biology, University of Birmingham, Edgbaston, Birmingham B15 2TT, UK; School of Biosciences, College of Life and Environmental Sciences, University of Birmingham, Edgbaston, Birmingham B15 2TT, UK; Birmingham Centre for Genome Biology, University of Birmingham, Edgbaston, Birmingham B15 2TT, UK; Division of Molecular and Cellular Function, School of Biological Sciences, Faculty of Biology, Medicine and Health, University of Manchester, Oxford Road, Manchester M13 9PT, UK

## Abstract

ELAV/Hu RNA-binding proteins are gene-specific regulators of alternative pre-mRNA processing. ELAV/Hu family proteins bind to short AU-rich motifs which are abundant in pre-mRNA, making it unclear how they achieve gene specificity. ELAV/Hu proteins multimerize, but how multimerization contributes to decode degenerate sequence environments remains uncertain. Here, we show that ELAV forms a saturable complex on extended RNA. Through phylogenomic instructed target analysis we identify the core binding motif U_5_N_2_U_3_, which is repeated in an extended binding site. Optimally spaced short U_5_N_2_U_3_ binding motifs are key for high-affinity binding in this minimal binding element. Binding strength correlates with ELAV-regulated alternative poly(A) site choice, which is physiologically relevant through regulation of the major ELAV target *ewg* in determining synapse numbers. We further identify a stem–loop secondary structure in the *ewg* binding site unwound upon ELAV binding at three distal U motifs. Base-pairing of U motifs prevents ELAV binding, but *N*^6^-methyladenosine (m^6^A) has little effect. Further, stem–loops are enriched in ELAV-regulated poly(A) sites. Additionally, ELAV can nucleate preferentially from 3′ to 5′. Hence, we identify a decisive mechanism for ELAV complex formation, addressing a fundamental gap in understanding how ELAV/Hu family proteins decode degenerate sequence spaces for gene-specific mRNA processing.

## Graphical Abstract

**Graphical Abstract:**
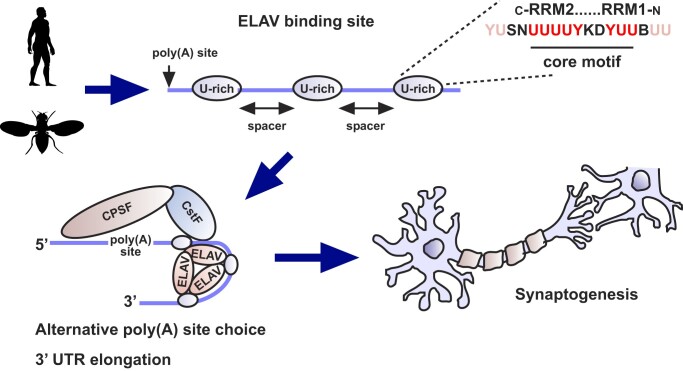

## Introduction

Alternative splicing and alternative 3′ end processing are general features of most eukaryotic genes to increase proteomic diversity and temporo-spatial control of gene expression (1–5). Key components for tight regulation of alternative mRNA processing are RNA-binding proteins which bind to dedicated sequence motifs to alter use of processing sites (6). SELEX (Systematic Evolution of Ligands by EXponential enrichment) experiments have revealed short recognition motifs for most RNA-binding proteins, but such motifs are found ubiquitously (3,7). Hence, it remains uncertain how RNA-binding proteins achieve gene specificity in a degenerate sequence environment.

RNA-binding proteins generally have a modular domain structure of one or more RNA-binding domains and one or more auxiliary domains (6,8,9). The most prevalent RNA-binding domain in eukaryotes is the RNA recognition motif (RRM) (6). A single RRM generally binds to 4–7 nucleotides, however multiple RRMs in conjunction do not add up to a unique binding motif (10,11).

ELAV/Hu family proteins are prototype RNA-binding proteins characterized by three RRMs, where RRMs one and two are separated by a flexible hinge region from the third RRM (12,13). There are four ELAV family members in humans (HuA/HuR, HuB/Hel-N1, HuC and HuD) and three in *Drosophila* (ELAV, RBP9 and FNE). Intriguingly, honeybees have only one ELAV, however it achieves molecular diversity through extensive alternative splicing (14).

ELAV has been shown to regulate gene-specific alternative splicing and 3′ end processing in *Drosophila*neurons (15–22). Initially, human ELAV/Hu family members have mostly been associated with cytoplasmic RNA-processing including regulating mRNA stability, localization and translation (23–27). However, ELAV/Hu family members shuttle between the nucleus and cytoplasm and can adopt various mRNA processing functions according to their localization in a concentration dependent manner (28–31).

Key insights into how ELAV achieves target-specific binding came from the characterization of its role in regulating *ewg* alternative splicing in the *Drosophila* nervous system (20,21,32). Here, ELAV binds in the proximity of an intronic poly(A) site and blocks 3′ end processing to allow for neuron-specific splicing of *ewg* (20), which is important to tune the number of synapses formed at neuromuscular junctions (33,34). Essential for ELAV to exert its function is binding to an extended binding site containing multiple U-rich motifs, but the binding site can diverge and still be functional as observed in distantly related *D. virilis* (32,35,36). On this extended binding site in *ewg*, ELAV forms a multimeric complex of about 700 kDa containing up to twelve ELAVs and one RNA (21,31). A key protein interaction domain in ELAV/Hu proteins has been mapped to RRM3, which is essential for dimerization (37–40).

Here, we show that the 700 kDa ELAV-RNA complex is saturated on the *ewg* binding site. Motif analysis in the *ewg* binding site, enhanced by phylogenomic sampling, reveals a core U_5_N_2_U_3_ binding motif. Through phylogenomic analysis of ELAV-regulated genes with alternative poly(A) site selection and ELAV binding assays, we find that distinct spacing of U_4_ motifs downstream of poly(A) cleavage sites is conserved between *Drosophila* species and in humans. Moreover, optimal spacing of U-rich motifs is essential for high-affinity ELAV binding. We further demonstrate that the level of alternative poly(A) site selection in ELAV-regulated genes depends on ELAV binding strength, which is underpinned by higher evolutionary conservation. Moreover, ELAV binding strength in its major target *ewg* directs the number of synapses made at neuromuscular junctions. In the *ewg* ELAV binding site we further identify a stem–loop structure which is unwound upon ELAV binding. Through sequence and secondary structure analysis we identify an enrichment of evolutionarily conserved *ewg*-like stem–loops in ELAV-regulate genes with alternative poly(A) site selection, that together with ELAV’s ability to preferentially nucleate from 3′ to 5′, facilitate target recognition. Our findings reveal essential sequence requirements for ELAV complex formation to overcome degeneracy of low complexity sequences for gene-specific regulation of alternative mRNA processing.

## Materials and methods

### Glycerol gradient centrifugation

Glycerol gradients were made by layering 200 μl of cold 30%, then 20% and 10% Glycerol in 0.5× TBE, 150 mM NaCl in the centrifugate tubes (5 × 41 mm Ultra-Clear, Beckmann 344 090) and stored overnight in at 4°C. The ELAV complex was assembled as in the EMSA protocol to a final volume of 30 μl (ELAV concentration of 2–3 μM determined on SDS-gel with BSA and ovalbumin standards) and loaded onto the gradient. The gradient was run on an ultra-centrifuge (Beckmann L-70) for 3.5 h at 38,000 rpm using the TST 55.5 swing out rotor (Kontron) with custom made Teflon adaptors. Once ultra-centrifugation was complete, 20 fractions of 25 μl were collected and 2 μl of each fraction was mixed with 8 μl formamide blue juice (formamide with 1 mM EDTA, 0.01% xylene and 0.01% bromophenol blue) and heat denatured for 90 s at 100°C and 2 μl were run on an 8% denaturing gel.

### RNA footprinting

Protein–RNA complexes were formed as for EMSAs in 9 μl total volume for 15 min at room temperature. Appropriately 1 μl diluted RNase (in 50 mM NaCl) was added and incubated for a further 15 min at room temperature. Micrococcal nuclease 1 mM CaCl_2_ and for RNase V1 1 mM MgCl_2_ was included in the assay. Reactions were quenched in stop buffer (100 mM Tris, pH 7.5, 10 mM EDTA, 1% SDS, 150 mM NaCl, 300 mM NaAc), phenol–chloroform extracted and ethanol precipitated.

### Electrophoretic mobility shift assays and cross-linking denaturing gels

Recombinant ELAV proteins were produced in *Escherichia coli* as GST-fusion proteins according to the manufacturer's instructions and cleaved off the GST-moiety with PrecissionProtease (Amersham), and stored in protease cleavage buffer after removal of the protease. Gel purified RNA in 50 μg/ml tRNA (Roche) and 1 U/μl RNasin (Roche) was heated for 5 min at 65°C, renatured at room temperature, then mixed with an equal volume of recombinant protein to a final concentration of 50 mM Tris-HCl pH 7.5, 60 mM NaCl, 25 μg/ml tRNA, 0.5 mM DTT, 50 μg/ml BSA (acetylated) in a total of 10 μl and incubated for 20 min at room temperature. Five μl mixed with 2 μl 50% glycerol was loaded on 4–6% (80:1 acrylamide/bisacrylamide) polyacrylamide gels and run at 4°C at 200 V in 0.5× TBE. Omitting EDTA had no effect on complex stability. Dye was loaded with input RNA.

The intensities of the free and bound RNA bands were quantified with Quantity One 1-D (Bio-Rad) as previously described (41). Fractions of bound RNA were calculated as the ratio of the intensity of the unbound band to the total intensity of the 0% bound fraction. To determine the dissociation constant (*K*_d_), the fraction bound was plotted against the protein concentration and the concentration of protein required to achieve 50% binding was determined by interpolation.

### Sequence retrieval and evolutionary analysis

Genomic sequences, phyloP27way data and alignments were retrieved from UCSC Genome Browser using the UCSC Table Browser sequence retrieval tool (42,43). Regions of 200 nucleotides upstream and downstream of cleavage sites were taken for each gene. RNA secondary structure predictions and free energy values were calculated using the RNA Folding Form in mfold (44). Phylogenetic information was obtained from Li *et al.* (45) and fly images were obtained from Flybase.

For U_4_ enrichment analysis, start positions of U_4_ motifs, downstream of the cleavage site for nucleotides 1–100 or 1–150, were located for each gene and assigned the value of 1 (where 0 signified absence of a U_4_ motif start position). The sum of all genes at each nucleotide position was calculated per gene group and a sliding window of five nucleotides centred around the first nucleotide was used to calculated accumulation of U_4_ motifs, from which heatmaps and scatterplots were generated. Individual U enrichment percentages were calculated per nucleotide and a sliding window of 11 nucleotides centred around the first nucleotide was used to generate scatterplots.

Sequence conservation of each nucleotide was calculated based on the average percentage of each nucleotide at each position in a 100-nucleotide region. Accumulation of nucleotide changes was quantified from phyloP27way data between species for analysed genes as previously described with an additional separation step for each nucleotide (46,47).

### Motif analysis

MEME-suite (version 5.4.1) was used to discover *de novo* motifs and probability matrices 200 nucleotides downstream of cleavage sites (48). Probability matrix scores were calculated as individual nucleotide positional values minus the sum of the remaining matrix values at that position, from which heatmaps were generated.

### Fly genetics and immunostaining of tissues

Fly crosses were maintained at 25°C in plastic vials containing 15 ml of a standard cornmeal/yeast-rich medium with a 12:12 h light–dark cycle. To analyse synapses at NMJs of third instar wandering larvae, *tcgER* transgenes were crossed to *ewg^l1^/FM7 GFP* females. Males from the progeny of this cross were then dissected in PBS, following fixation with Bouin's solution (Sigma-Aldrich, HT10132) for 5 min. Tissues were washed in PBT [PBS with 0.2% BSA and 0.1% TritonTM X-100 (Sigma, T8787)] 3 × 15 min. Samples were incubated overnight at 4°C with primary antibodies rabbit anti-HRP (1:250, 323 005 021, Jackson ImmunoResearch) and mouse anti-NC82 (1:100, DSHB), followed by secondary antibodies conjugated with (Alexa Fluor 488 or Alexa Fluor 546) again overnight at 4°C. NMJs were mounted in Vectashield (Vector Labs), scanned with Zeiss Imager.M2 ApoTome.2 and processed using FIJI.

## Results

### ELAV forms a saturable complex on extended RNA

**Figure 1.**
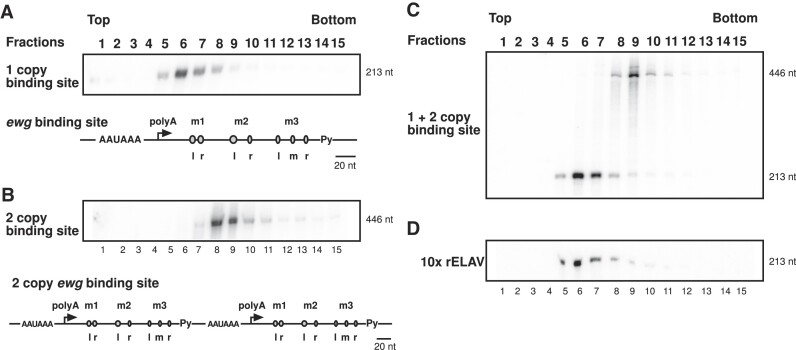
ELAV forms a saturable complex on *ewg* RNA. (A–D) Glycerol gradient centrifugation fractions from top to bottom of recombinant ELAV with (**A**) 1 copy ELAV binding site RNA from *ewg*, (**B**) 2 copy ELAV binding site RNA from *ewg*, (**C**) 1 and 2 mixed copy ELAV binding site RNA from *ewg* and (**D**) 1 copy ELAV binding site RNA from *ewg* with a 10-fold increased ELAV concentration. After RNA extraction, fractions were run on 8% denaturing gels. 1 and 2 copy binding site RNAs ran in distinctly different positions (A–C), while increasing the concentration of ELAV had no effect on complex size (D). As detailed in A, the ELAV binding site in *ewg* harbours three U-rich tandem motifs termed m1, m2 and a triple U-motif in m3. RNA substrates are depicted in (A) and (B) with m1, m2, m3 and respectively left (l), right (r) and middle (m) U-rich sequences highlighted, described in Soller and White (21).

Before analysing ELAV binding requirements, we first determined whether the 700 kDa ELAV-RNA complex is saturable. We employed glycerol gradient ultracentrifugation with synthetic RNAs of either one or two copies of the *ewg* binding site (which harbours three U-rich motifs labelled m1, m2 and m3 in Figure 1A and B) of 213 or 446 nucleotides, respectively (20,21). When combining these two substrate RNAs for ELAV binding and complex formation, we observed that glycerol gradient centrifugation separates two ELAV complexes after centrifugation, RNA extraction and separation on denaturing polyacrylamide gels (Figure 1A–C). To test whether ELAV forms a saturable complex, we increased the amount of ELAV 10-fold for binding to substrate RNA containing one copy of the *ewg* binding site and observed the same sized complex (Figure 1D). This result demonstrates that the 700 kDa ELAV complex is saturated and that complex formation is not due to concentration dependent aggregation.

### ELAV binding sites at regulated poly(A) sites contain three spaced and evolutionarily conserved U-rich motifs downstream of the cleavage site

**Figure 2.**
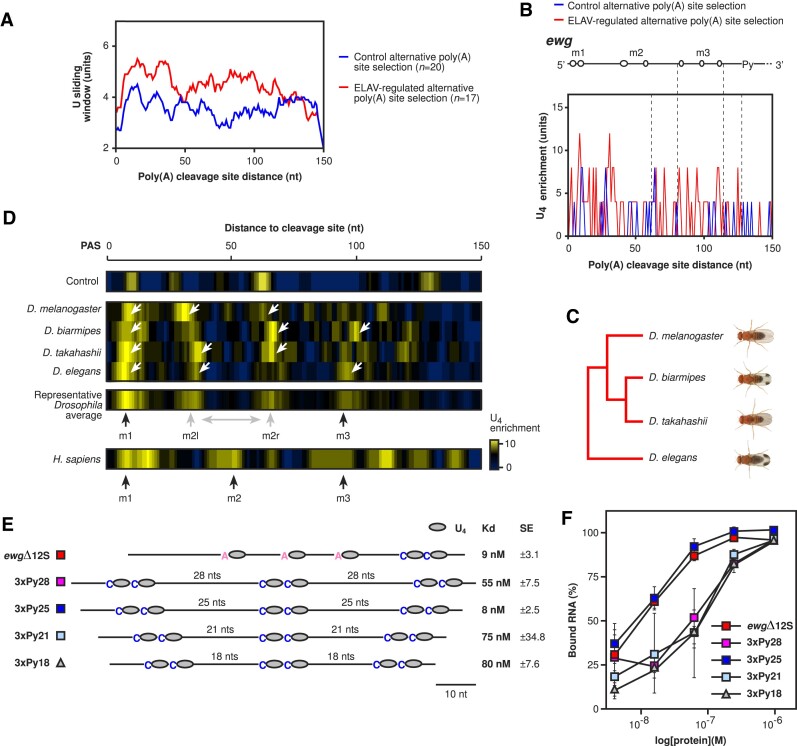
Spaced U-rich motif architecture is evolutionarily conserved between *Drosophila* and humans. (**A** and **B**) Comparative U and U_4_ enrichment sliding window scores calculated for 150 nucleotides downstream of control (blue) and ELAV-regulated (red) alternative poly(A) site selection gene cleavage sites in *D. melanogaster*. Positions of relevant high-affinity *ewg* ELAV binding sites are indicated spatially in schematics above (C). (**C**) Phylogeny of the *Drosophila* species analysed (*D. melanogaster*, *D. biarmipes*, *D. takahashii* and *D. elegans*). (**D**) U_4_ enrichment in control and ELAV-regulated alternative poly(A) site selection genes compared between *D. melanogaster*, *D. biarmipes*, *D. takahashii*, *D. elegans* and *H. sapiens* for the 150-nucleotide region downstream of poly(A) cleavage sites. Enriched motif positions are indicated with black and grey arrows labelled as m1, m2 (m2l: left and m2r: right) and m3. Predominant motif positions were calculated based on the highest three spatial conservation scores between the four motifs within the *Drosophila* average range (Supplementary Dataset 1). (**E** and **F**) Schematic depiction of the *ewg* and synthetic RNAs used for EMSA analysis of motif spacing effects on ELAV binding affinity (E). The *ewg*Δ12S control RNA (only *ewg*, no vector sequence as in *ewg*Δ12 (21)) was used as a high-affinity positive control. EMSA results were quantified and plotted (F). The average and standard error at each concentration were calculated from at least two biological replicates (E). *K*_d_ values and standard deviation are shown in (F).

Since ELAV complex formation on the minimal *ewg* binding site requires multiple spaced U-rich motifs (21,32), we first analysed U enrichment in the 150 nucleotides downstream of the cleavage site in ELAV-regulated genes with alternative poly(A) site choice (17 genes, Supplementary Dataset 1) compared to non-ELAV regulated genes (20 genes, Supplementary Dataset 1) (49,50). As anticipated, ELAV targets are more U-rich, particularly within the first 50 nucleotides after the cleavage site (Figure 2A). To analyse ELAV binding sites for defined patterns of U enrichment, we used a scanning window of four Us to determine positional enrichment. Here, we observed U enrichment in a regular spaced pattern in *Drosophila melanogaster* ELAV targets compared to control genes (Figure 2B). Interestingly, this U enrichment pattern aligns with the *ewg* ELAV binding site designated m1, m2 and m3 (Figure 2B, top).

To potentiate this motif analysis, we employed phylogenomic instructed analysis among closely related *Drosophila* species (*D. melanogaster*, *D. biarmipes*, *D. takahashii* and *D. elegans*) (Figure 2C) within these 17 ELAV targets, which have strictly conserved gene structures. Strikingly, this analysis confirmed a conserved motif immediately after the cleavage site (m1) and three major additional U-rich motifs with regular spacing (Figure 2D, m2l, m2r and m3 as in Figure 1A). From these three additional motifs, two (m2l, m2r) are flexible between species (Figure 2D, Supplementary Dataset 1).

RRMs of *Drosophila* and human ELAV/Hu proteins are highly conserved (26). Therefore, we anticipated that human HuR, which is closest to *Drosophila* ELAV and can rescue lethality in ELAV mutants (31), would adopt a similar motif spacing preference in its targets. To be able to compare binding of Hu proteins to our ELAV data, we repeated our analysis with a random subset of 20 human genes regulated by all four Hu proteins after RNAi in human iPSC-neurons (51). Strikingly, we also observed three regularly spaced U-rich motifs (Figure 2D). Intriguingly, left and right boundaries were exactly at the same position as in *Drosophila*, suggesting a conserved extended binding site of around 100 nucleotides containing three U-rich motifs (Figure 2D). However, the position of the middle motif seems to be flexible as deduced from comparison among different *Drosophila* species (Figure 2D).

A key feature for high-affinity binding of ELAV in the *ewg* gene are three U-rich motifs in an extended binding site (21), but whether spacing of these U-rich motifs affects ELAV binding has not been tested. Hence, we employed EMSAs with synthetic RNAs containing three U-rich motifs (CU_4_CU_4_). This motif is present in the distal part of the ELAV binding site in *ewg* and mutating this motif results in the strongest loss of affinity (21). As spacers, we used adenosines ranging from 18 to 28 nucleotides (A_18_, A_21_, A_25_, A_28_) (Figure 2E, Supplementary Figure S1). These experiments revealed the highest affinity for a spacer of 25 nucleotides and was comparable to the *ewg* ELAV binding site substrate *ewg*Δ12S (Figure 2F, *ewg*Δ12S is devoid of nucleotides not protected in footprinting experiments shown later in Figure 6).

SELEX experiments with 7-mers revealed a U_2_N_2_U_3_ motif for ELAV/Hu family proteins (7). In an initial attempt to evaluate ELAV binding to RNA substrates containing the SELEX motif we used six U_2_A_2_U_3_ motifs spaced by six As. Intriguingly, we did not observe ELAV binding to this substrate (6xSM-A), but binding was restored when we used A_3_C_3_ spacers (6xSM-AC, Supplementary Figure S2). When we analysed the secondary structures of these two substrates, we noticed that all Us were base-paired in 6xSM-A indicating that ELAV can only bind if Us are not base-paired (Supplementary Figure S2). It has been suggested that *N^6^*-methyladenosine (m^6^A) can disrupt base-pairing (52,53). However, when m^6^A was incorporated into the 6xSM-A substrate RNA, binding was not restored (Supplementary Figure S2).

### ELAV binding sites distal of alternative poly(A) sites contain a conserved extended binding motif

**Figure 3.**
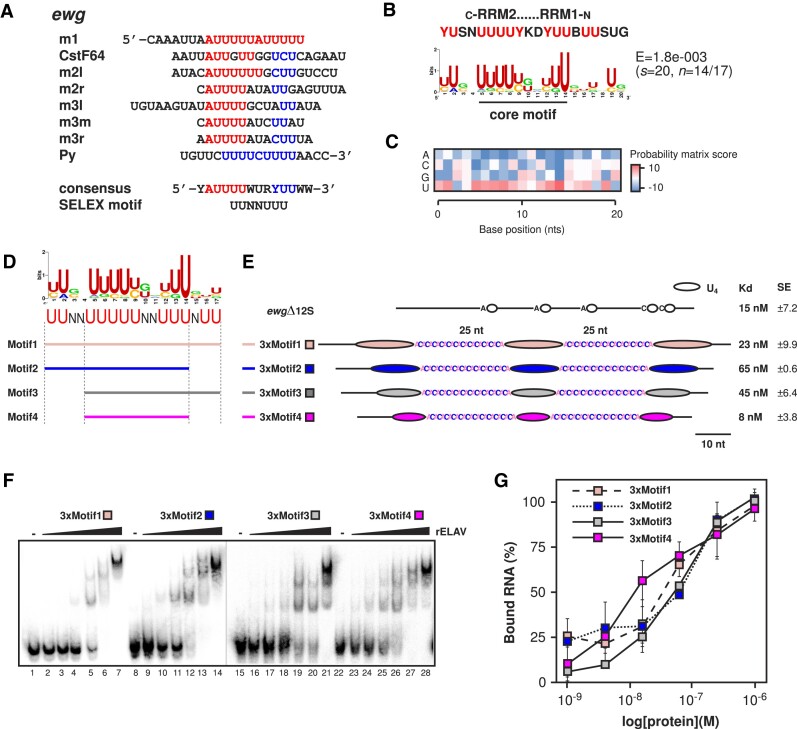
ELAV requires a conserved, defined U-rich motif for high-affinity binding. (**A**) Alignment of consecutive AU4-6 motifs from the *ewg* ELAV binding site. AU4-6 motifs are in red and the PyU2 motif is blue. The CstF64 binding site likely represents a suboptimal AU4-6 motif. A putative consensus sequence for RRM1 and RRM2 is indicated at the bottom. Sequence labels are in reference to *ewg* U-rich sequence positions, shown in Figure 1A and B, and detailed in Soller and White, 2005 (21). (**B** and **C**) Comparative *de novo* extended motif found from alignment of the 200-nucleotide poly(A) cleavage site downstream regions of 17 ELAV-regulated gene targets (see Figure 7B for the full list of genes). A putative consensus sequence for RRM1 and RRM2 is indicated at the top. (**D** and **E**) Consensus motif analysis of the analysed ELAV targets with schematic depiction of deletion mutagenesis motifs generated to test core components of the extended ELAV binding site, labelled Motif1 to 4 (D). RNA substrate schematics are indicated, incorporating optimal spacing and unpairing spacer sequences (E). The *ewg*Δ12S control RNA was used as a high-affinity positive control. (**F** and **G**) EMSA analysis of the synthetic RNA substrates (F) and quantification of ELAV complex formation (G). The average and standard error at each concentration were calculated from at least three biological replicates (G). *K*_d_ values and standard deviation are shown in (E).

The *ewg* ELAV binding site harbours a six-fold repeated AU_4_ motif, that upon alignment revealed an additional conserved YU_2_ motif (Y for pyrimidine) three nucleotides distal of the AU_4-6_ motif resulting in an extended YAU_4_WURYU_2_W_2_ motif (Figure 3A, W: A or U, R: purine). This extended motif also endorses the ELAV/Hu SELEX motif (7). Moreover, co-crystallization experiments of the first two RRMs of HuD, or Sex-lethal (Sxl), the closest relative of ELAV in *Drosophila* (11,54), with U-rich RNAs align with this extended motif.

Motif analysis of ELAV target genes with alternative poly(A) site selection revealed a core motif U_5_N_2_U_3_, though this motif lacked the preceding A found in the *ewg* ELAV binding site (Figure 3B and C) (49,50). In addition to this core motif, two Us distal and two Us proximal are conserved indicating the following motif U_2_N_2_U_5_N_2_U_3_NU_2_ from phylogenomic comparisons (Figure 3B and C).

Analysis of RNA co-purifying with recombinant ELAV from *E. coli* is not characteristically U-rich and substantially differs from the *Drosophila* motif. Moreover, *in vitro* binding of ELAV was not enhanced after RNase removal of RNA derived from *E. coli*, indicating that this is not a competitor (Supplementary Figure S3).

### ELAV requires a defined U-rich motif in an optimally spaced context for high-affinity binding

Next, to evaluate how individual components of the extend motif contribute to ELAV binding, we employed EMSAs with synthetic RNAs containing three motifs spaced by 25 nucleotides of AC sequences (Figure 3D and E). The tested motifs were the full-length motif (Motif1), the core motif with the distal extension (Motif2), the core motif with the proximal extension (Motif3), and the core motif itself (Motif4). Here, ELAV had a high affinity for RNAs with three full motifs (3xMotif1, *K*_d_: 23 nM) or three core motifs (3xMotif4, *K*_d_: 8 nM), which is comparable to the ELAV *ewg* binding site RNA *ewg*Δ12S (*K*_d_: 15 nM, Figure 3F and G). Removing flanking U-rich sequences either on either side resulted in reduced affinity (3xMotif2, *K*_d_: 65 nM and 3xMotif3, *K*_d_: 45 nM) (Figure 3F and G).

### Extent of ELAV-regulated alternative poly(A) site selection depends on a high-affinity binding site

**Figure 4.**
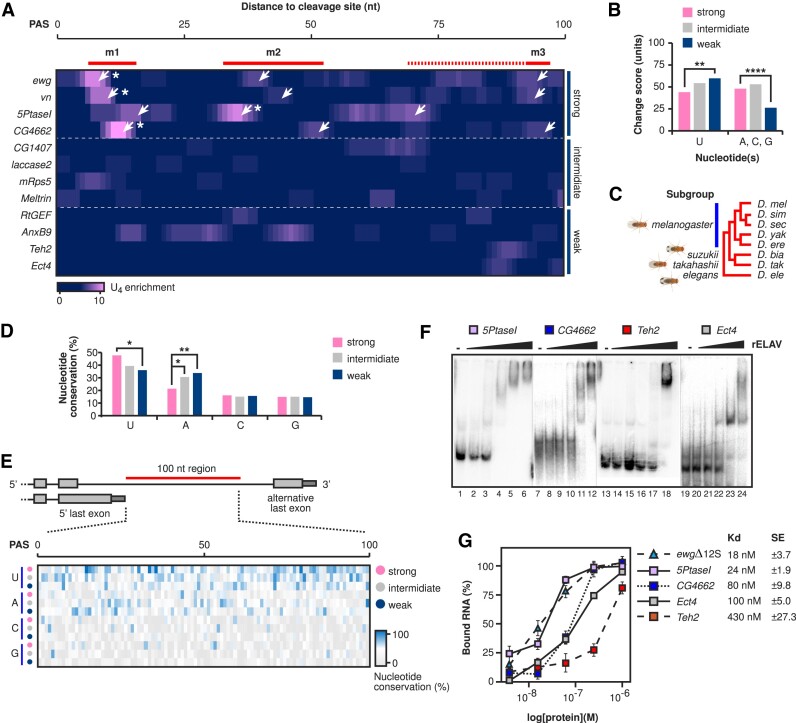
ELAV-regulated alternative poly(A) site selection depends on a high-affinity binding site. (**A**) U_4_ enrichment sliding window scores calculated for 100 nucleotides downstream of ELAV-regulated genes with alternative poly(A) site selection. Genes are separated into three categories: strong (>4-fold change), intermediate (<4-fold, >2-fold change) and weak (<2-fold change) based on their last exon usage fold change between wild type and ELAV null mutants (49,50). Motif positions relative to those shown in Figure 2D are labelled (m1, m2, m3). (**B–E**) Analysis of sequence change accumulation using phyloP scores (B) and evolutionary conservation (D and E) between the closely related *melanogaster* subgroup, *D. biarmipes* (*suzukii* subgroup), *D. takahashii* (*takahashii* subgroup) and *D. elegans* (*elegans* subgroup) (C) for each nucleotide type (A, C, G, U) in each fold change category. Statistically significant differences from chi squared tests are indicated by asterisks (Bonferroni corrected *P ≤ 0.05, **P ≤ 0.01, ****P ≤ 0.0001). (**F** and **G**) EMSA analysis of the synthetic RNA substrates made for *5PtaseI*, *CG4662*, *Teh2* and *Ect4* (F) and quantification of ELAV complex formation (G). The average and standard error at each concentration were calculated from at least two biological replicates (G). *K*_d_ values and standard deviation are shown in (G).

To test whether the strength of ELAV regulation in alternative poly(A) site selection is accompanied by optimal spacing of U-rich motifs, we analysed spatial conservation of U-rich sequences in ELAV-target genes. We based our gene choices on fold change scores of alternative last exon usage between wild type and *elav* null mutants using three categories, strong (>4-fold change), intermediate (<4-fold, >2-fold change) and weak (<2-fold change, Figure 4A) (49,50). Analysis of spatial phylogenomic conservation of U-rich sequences in the three categories revealed substantial U enrichment for the strong category at positions m1, m2 and m3 with variability between positions m2l, m2r and m3, as previously described (Figure 4A, see also Figure 2D).

Analysing the accumulation of sequence changes using phyloP scores revealed that changes to Us are significantly reduced in strongly ELAV-regulated alternative poly(A) sites, while for all other nucleotides (A, C, G) we identified a bias in weak sites (Figure 4B). Next, we performed a more detailed analysis of the evolutionary conservation between the *melanogaster* subgroup (*D. melanogaster*, *D. simulans*, *D. sechellia*, *D. yakuba* and *D. erecta*), *D. biarmipes* (*suzukii* subgroup), *D. takahashii* (*takahashii* subgroup) and *D. elegans* (*elegans* subgroup) for each nucleotide (A, C, G, U) according to the strength of poly(A) site selection (Figure 4C-E). Conservation of U’s significantly correlated with suppression of poly(A) site selection, while the opposite was the case for As (Figure 4C-E). No significant change was observed for the conservation of Cs and Gs (Figure 4C-E).

To test whether ELAV-regulated poly(A) site selection is dependent on ELAV binding affinity, we chose genes with strong or weak effects on poly(A) site selection in the absence of ELAV by selecting genes with strong effects present in multiple datasets (49,50,55). We generated *in vitro* transcribed RNAs for the 100-nucleotide region downstream of the cleavage site and analysed binding affinities using EMSAs (Figure 4F and G). The strong poly(A) site choice category genes *5PtaseI* and *CG4662* both formed full complexes and showed high binding affinities with *K*_d_s of 24 and 80 nM, respectively (Figure 4G). Conversely, the weak poly(A) site choice category genes *Teh2* and *Ect4* had lower affinities with *K*_d_s of 430 and 100 nM, respectively (Figure 4G). In addition, *Ect4* formed only an intermediary complex at the highest ELAV concentration (Figure 4F, well 24).

### ELAV binding directs synapse formation

**Figure 5.**
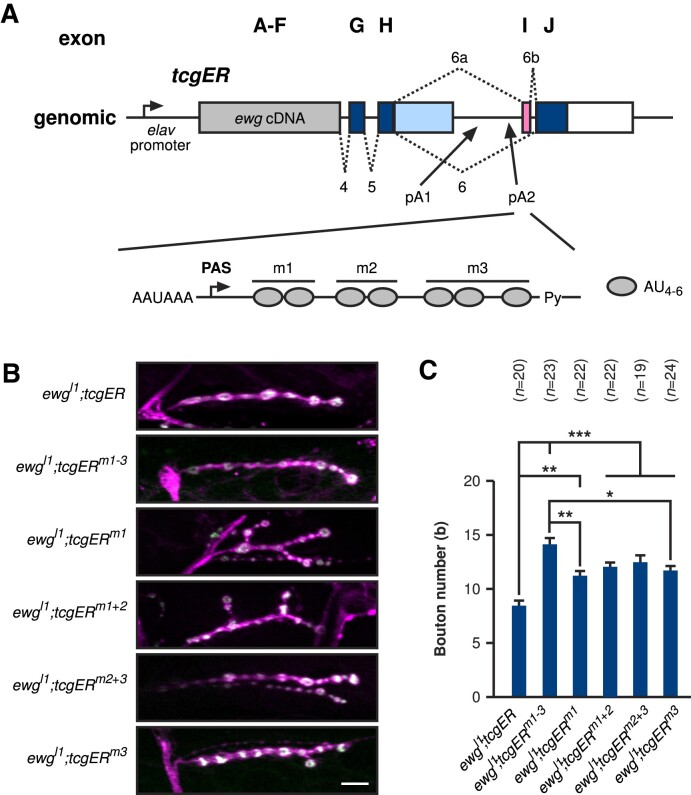
ELAV binding is required for synapse formation. (**A**) Schematic of the *tcgER ewg* transgenic rescue construct. EWG is expressed in the nervous system from a chimeric cDNA/genomic construct. The pA2 ELAV binding site is detailed with description of U-rich motifs m1, m2 and m3. Mutants were generated for m1-3, m1, m1-2, m2-3, and m3 as described in Soller and White (2005). (B, C) Representative images (**B**) and quantification (**C**) of NMJs from muscle 13 synapses for the analysed genotypes in an *ewg* null background (*ewg^l1^*). Motor neurons are stained with anti-HRP (magenta) and synaptic boutons with anti-NC82 (green, white in the merged picture). Statistically significant differences from ANOVA with Tukey's multiple comparisons correction are indicated by asterisks (*P ≤ 0.05, **P ≤ 0.01, ***P ≤ 0.001 adjusted *P* values). The scale bar (B) is 10 μm.

Next, we analysed whether binding strength impacts on synapse formation at third instar neuromuscular junctions (NMJs) directed by ELAV mediated suppression of poly(A) site choice in the last intron of *ewg*, resulting in splicing of the last intron and expression of EWG protein (16,56). We chose *ewg* because it is a major target of ELAV indicated from rescue *elav* mutants by *ewg* cDNAs (33,34). For these experiments, we used transgenes of the *tcgER* rescue construct in an *ewg^l1^* null background. In this transgene, we had introduced mutations in U-rich motifs m1, m2 and m3 individually and in combinations, which lead to a reduction of *ewg* last intron splicing depending on the number of mutations introduced in the extended ELAV binding site (Figure 5) (21).

Compared to the control, mutations in all U-rich motifs (m1-3) in the ELAV binding site in the *ewg* regulated intron resulted in an increase in the number of synaptic boutons consistent with EWG’s cell-autonomous role in limiting synaptic growth (Figure 5B and C) (33,34). Likewise, mutations in individual U-motifs or combinations thereof, were less effective in restricting synapse formation (Figure 5B and C).

### The minimal ELAV binding site in *ewg* contains a stem–loop structure which is unwound upon binding

The affinity for ELAV in the *ewg* binding site is higher in the proximal part termed *ewg*Δ12 (21,31). Since ELAV binding could be linked to RNA secondary structure, we analysed whether the ELAV binding site from *ewg* adopts a secondary structure. Mfold predicts that *ewg*Δ12 adopts a stem–loop structure.

**Figure 6.**
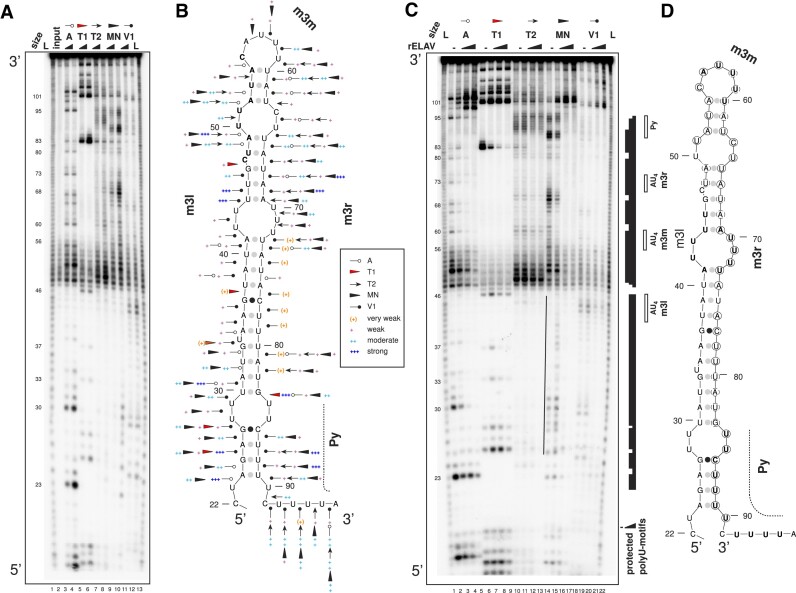
The minimal ELAV binding site in *ewg* contains a stem–loop structure which is unwound upon binding. (**A**) Enzymatic probing of ewg RNA pA2-IΔ12 for secondary structure and RNase footprinting of ELAV. (A) Enzymatic probing of *ewg* RNA pA2-IΔ12. Sensitivity of 5′ ^32^P labelled substrate RNA (100 pM) was tested for two concentrations of RNases A, T1, T2, V1 and micrococcal nuclease (MN) as indicated on top. L: single nucleotide ladder. RNase A cleaves preferentially after single stranded pyrimidines but can also cleave in helical regions. RNase T1 cleaves after single stranded G residues. RNase T2 cleaves single stranded regions with preference for A residues. Micrococcal nuclease cleaves single stranded regions. RNase V1 cleaves double stranded regions but can also cleave helical single strands. (**B**) Summary of enzymatic probing results from at least three experiments for each RNase. Secondary structure predictions were obtained with mfold and adjusted to experimental data. Nucleotides sensitive to hydrolysis are bold (nucleotides 47–55). The polypyrimidine tract (Py) is indicated by a dotted line. RNase cut strength is shown as (+) very weak, + weak, ++ moderate, and +++ strong. (**C**) RNase footprinting of *ewg* RNA pA2-IΔ12 in the presence of increasing amounts of ELAV (0.2, 0.8 and 3.2 μM) or in the absence of ELAV (–). Sequence landmarks (polyU motifs: m3l, m3m, m3r and Py) and protected sites (black bars) are indicated on the right. The black line to the left of the micrococcal nuclease series (MN, lanes 14–17) indicates nucleotides 25–46 which become nuclease-sensitive at the lowest ELAV concentration (0.2 μM). L: single nucleotide ladder. (**D**) Schematic summary of footprinting results with 0.2 μM ELAV in the context of pA2-IΔ12 secondary structure as determined in (A) and (B). Strongly protected nucleotides are circled and weak protection is indicated with broken circles. polyU motifs are bold (m3l, m3m, m3r and Py). The polypyrimidine tract is also indicated by a dotted line.

Enzymatic probing of 5′ end-labelled RNA *ewg*Δ12 largely overlaps with the predicted stem–loop secondary structure (Figure 6A, lanes 3–12, and B). Minor discrepancies can be attributed to ‘breathing’ of paired regions as seen by weak cleavage of single strand specific RNases (A, T1, T2 and micrococcal nuclease). RNase V1 detects paired regions, and the cleavage pattern is therefore complementary to the pattern generated by single strand specific RNases. In addition, RNase V1 can also cleave single stranded regions, if a helical configuration extends over 4–6 nucleotides that serve as a recognition sequence (57). Extended single-stranded helical configuration of the m3 region is indicated by RNase V1 cleavage at the top of the stem–loop structure (m3m element, Figure 6B) as well as in the 3′ half of the polypyrimidine tract (Py). In addition, a ladder like appearance of untreated RNA between nucleotides 47 and 55 (Figure 6A, lane 2) indicates sensitivity to hydrolysis, most likely superimposed by tension through a helical configuration.

Next, we performed RNA footprinting experiments in the presence of increasing ELAV concentrations on 5′ end-labelled RNA *ewg*Δ12 to further delineate the binding site of the ELAV complex (Figure 6C and D). At the lowest ELAV concentration the most pronounced protections are seen between nucleotides 48 and 98 (Figure 6C, lanes 3, 7, 11, 15, 19), which include m3m and m3r elements and part of the polypyrimidine tract. Increasing ELAV concentrations results in further protection including nucleotides 22–45 (m3l element). However, the control flanking vector sequence (1–21 and 105–121) is mostly not protected or becomes sensitive to nuclease digestion with increasing ELAV concentrations, confirming that changes in RNase protection originate from ELAV binding (Figure 6C, see in the 3′ region from nucleotide 98 onwards, e.g. lanes 4 and 5).

Remarkably, the left half of the stem–loop structure (nucleotides 24–45) showed sensitivity to RNase digestion at lower ELAV concentrations compared to the absence of ELAV. This could result from the initiation of the ELAV complex on single stranded RNA in the right half of the stem–loop structure (Figure 6C, lanes 3, 7, 11, 15 and 19, and circled nucleotides in Figure 6D show protected nucleotides at the lowest ELAV concentration). Hence, the observed secondary structure could support initiation of ELAV complex formation.

Next, we performed evolutionary analysis of the *ewg* stem–loop for complementary base pair changes between closely related *Drosophila* species of the *melanogaster* subgroup (*D. melanogaster*, *D. simulans*, *D. sechellia*, *D. yakuba* and *D. erecta*) (Supplementary Figure S4A and B). In comparison to *D. melanogaster*, this analysis revealed that base changes which maintain pairing in the *ewg* stem–loop were present in three species (*D. simulans*, *D. yakuba* and *D. erecta*), while few non-complementary (*D. simulans* and *D. erecta*) and unpaired (*D. erecta*) base changes were also observed (Supplementary Figure S4A and B).

### ELAV-regulated genes with alternative poly(A) site selection preferentially contain stem–loop secondary structures in the ELAV binding site

**Figure 7.**
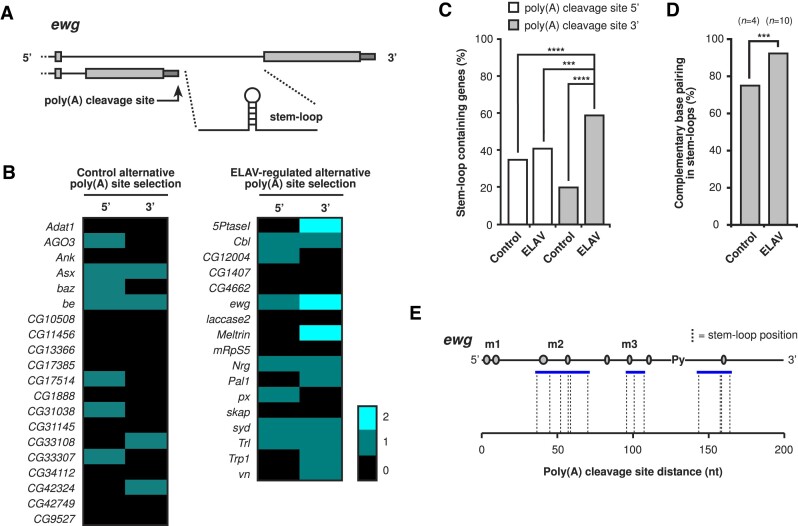
ELAV-regulated alternative poly(A) site selection gene targets preferentially contain stem–loop secondary structures. (**A**) Schematic of the downstream cleavage site region analysed for secondary structures in ELAV-regulated alternative poly(A) site selection genes, with *ewg* as an example. (**B** and **C**) Quantification of distinct *ewg-*like stem–loop structures in 200-nucleotide 5′ and 3′ regions of the control group and in ELAV targets for individual targets and as an overall percentage. Statistically significant differences from non-parametric chi-squared tests are indicated by asterisks (***P ≤ 0.001, ****P ≤ 0.0001 following Bonferroni correction). (**D**) Quantification of complimentary base pair changes in poly(A) cleavage site downstream regions of control and ELAV regulated genes. Complementary changes were classified as a nucleotide replacement required for stem–loop pairing between two species which maintained pairing. Statistically significant differences from non-parametric chi-squared tests are indicated by asterisks (***P ≤ 0.001 following Bonferroni correction). (**E**) Positions of stem–loops from ELAV target genes mapped onto the *ewg* ELAV binding site.

To identify whether stem–loops are a general feature of ELAV binding sites, we analysed genes with alternative poly(A) site selection regulated by ELAV with the same gene structure as *ewg*, e.g. a regulated poly(A) site in an intron (Figure 7A and B, 17 genes) (49,50). As a control group, we took 20 representative genes with the same gene structure as *ewg* which are not ELAV regulated (Figure 7B) (49,50). For this secondary structure analysis, we took a 200-nucleotide region downstream of the cleavage site. As additional controls, we also took 200-nucleotide regions upstream of cleavage sites for both the control group and ELAV targets. We then analysed the RNA secondary structures for stem–loops with similarity to that of the *ewg* ELAV binding site. Our criteria specifically identified stem–loops between 60 and 200 nucleotides in length, and with no additional loops protruding from the primary loop (Figure 6A and B). Examples of stem–loops selected based on these criteria are shown in Supplementary Figure S5A and C. Here, we found that genes with alternative poly(A) site selection regulated by ELAV were significantly enriched in this distinct stem–loop type compared to all control groups (Figure 7C). When analysing predicted free energy of the stem–loops (see Supplementary Figure S5B and D for base pair level stem–loop-specific examples), we found no significant changes between stem–loops enriched in the proximal part of the ELAV binding site compared to all control groups (Supplementary Figure S6).

To identify whether stem–loops in ELAV targets are conserved between closely related *Drosophila* species (*D. melanogaster*, *D. simulans*, *D. sechellia*, *D. yakuba* and *D. erecta*) we quantified complimentary base pair changes that maintain pairing between at least two species. Here, we found that ELAV targets had significantly more complimentary base changes compared to the control (Figure 7D). Further, we analysed the localization of the stem–loops relative to the cleavage site. We observed three positions for preferred stem–loop localization, with the majority being ∼50 nucleotides from the cleavage site (Figure 7E).

### ELAV nucleates from a high-affinity binding site preferentially from 3′ to 5′

**Figure 8.**
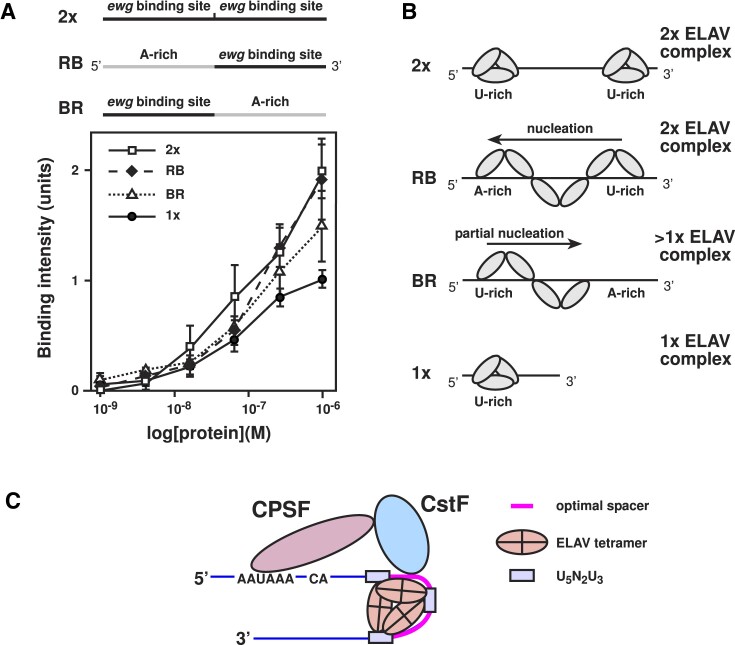
ELAV nucleates from a high-affinity binding site preferentially from 3′ to 5′. (**A**) Quantification of ELAV-RNA binding intensity (arbitrary units) for each of the cross-linked RNA variants run on denaturing gels. The 2× and 1× RNAs form regular complexes around the sizes of 1× and 2× ELAV complexes. The RB RNA (antisense site in the 5′ and a U-rich binding site in the 3′) formed a full 2× sized complex at high concentrations. The BR RNA (U-rich binding site in the 5′ and an antisense site in the 3′) formed an intermediary sized complex between 1× and 2× in size. (**B**) Schematic of the results from quantification of cross-linking gels showing the predicted mode by which ELAV forms larger complexes on single copy binding site RNA through nucleation with a directional 3′ to 5′ preference (shown through BR and RB) preference. (**C**) Schematic of CPSF, CstF, and ELAV binding to an ELAV target pre-mRNA with optimally spaced U-rich motifs. CPSF and CstF co-operative binding with ELAV in the *ewg* regulated poly(A) site selection could limit ELAV spreading via nucleation.

In the *ewg* ELAV binding site, the polypyrimidine tract of the adjacent exon serves as high-affinity binding site to initiate ELAV complex formation (21). In analogy to hnRNPA1 which can nucleate from a high-affinity binding site into neighbouring sequence (58–60), we wanted to test whether ELAV can nucleate as well. For this purpose, we made substrate RNAs containing an A-rich sequence either 5′ (RB) or 3′ (BR) of the *ewg* ELAV binding site (Figure 8A). As an A-rich sequence we used the reverse anti-sense sequence of the *ewg* ELAV binding site, which ELAV does not bind (20). Also, because it is the reverse sequence, it does not form a completely base-paired dsRNA.

Because electrophoretic mobility shift assays are limited in resolving such large complexes, we used in solution UV-crosslinking mediated ^32^P label-transfer from the RNA to ELAV followed by separation of ELAV on SDS gels to quantify RNA binding.

Compared to the one and two copy binding site controls, RB bound the same amount of RNA as the 2× complex despite possessing a single binding site (Figure 8A). In contrast, BR bound less RNA as the 2× complex, but more than the single complex (Figure 8A and B). These results indicate that ELAV can nucleate bidirectionally, but with a preference for the 3′ to 5′ direction. In this context, co-operative binding of CPSF and CstF together with ELAV in the *ewg* regulated poly(A) site likely serves to limit ELAV spreading (Figure 8C) (20).

## Discussion

ELAV/Hu RNA-binding proteins are highly conserved, but how gene-specific regulation is achieved through binding to short U-rich motifs that are common in introns and untranslated sequences remains uncertain. Our finding that ELAV forms a saturable complex on target RNA provides a mechanistic framework to explain the principles for its gene-specific regulation. To overcome limitations of SELEX-experiments, we employ phylogenomic analysis of ELAV target sequences and identify key architectural features from evolutionary conservation. In particular, we identify a U-rich core ELAV binding motif and find that optimal spacing of three core motifs provides the molecular basis for high-affinity binding and complex formation. Further, we demonstrate that strong ELAV binding correlates with increased regulation of alternative poly(A) site selection. Subsequently, using the ELAV target *ewg*, we show that strong ELAV binding is required for restricting synapse formation. Moreover, our analysis of RNA secondary structure in the *ewg* ELAV binding site together with evolutionary conservation of such stem–loop structures suggest a role in initiating ELAV binding to single-stranded RNA to instruct complex formation from unpaired U-rich motifs through ELAV’s capacity to nucleate into non-U-rich sequences.

### Optimally spaced motifs provide the molecular framework for ELAV complex formation and gene-specific regulation

ELAV regulation of 3′ end processing was first identified for the *ewg* gene and is now a recognized core function of ELAV/Hu proteins, also leading to extension of 3′ UTRs of many neuronal genes (20,50,61,62). Such a set of genes regulated in the same way provides a unique resource for detailed sequence comparisons and is potentiated by expanding into phylogenomic analysis using the genomes of many sequenced species. Through this phylogenomic analysis we identified a core consensus motif for ELAV binding. Moreover, this analysis revealed presence of three U-rich motifs in an extended binding site that require optimal spacing for highest affinity in *in vitro* binding assays. Molecular modelling has been used to enhance binding predictions for PTB from CLIP data (63). Here, we add an additional layer of information from evolutionary conservation to the analysis of *in vivo* binding of RNA-binding proteins which will help to make predictions more accurate.

Human HuR can functionally substitute *Drosophila* ELAV, and accordingly we find the architecture of the binding site conserved, particularly in the position of the two flanking motifs (31). However, we observed differences in the position of the middle motif, which also seemed to be species specific and could reflect a differential outcome from speciation processes. In addition, analysis of the human Hu binding site revealed three additional U-rich motifs distal from the poly(A) site to make up a binding site of about 150 nucleotides, which is the length initially characterized in *ewg*. Likewise, in this initial analysis we also noted six U-rich motifs. It is possible, that the ELAV complex can adopt two different conformations, a dense configuration on a minimal binding site of about 100 nucleotides and a relaxed conformation on about 150 nucleotides. Analysing the sequence requirements for the relaxed position in the proximal part is difficult because our sequence analysis requires an anchor point to counteract flexibility, which is the cleavage site on the distal side.

Evidence for the relevance of ELAV complex formation *in vivo* comes from the study of protein-protein interactions and the analysis of sequence requirements for ELAV dependence in the *Drosophila ewg* gene (35,36). Here, mutations in all U-rich sequences are required to completely abolish ELAV dependence (21). This finding is further substantiated through the analysis of the *D. virilis* ELAV binding site in *ewg* (32). The sequence of this site has massively diverged, yet the RNA-binding region of ELAV is identical in both species. Accordingly, the *D. virilis* site is fully functional in *D. melanogaster* and only mutation of all U-rich motifs abolishes ELAV regulation (32). Visualization of ELAV/Hu proteins in cells and *Drosophila* neurons revealed a granular appearance (23,64). Particularly in *Drosophila* ELAV subcellular localization to highly concentrated spots and webs has been observed (65), but for a complex of twelve ELAVs, analysis of the complex stoichiometry *in vivo*, as has been done for PTB, poses a challenge (66).

A major function of ELAV/Hu RNA-binding proteins is suppression of poly(A) site choice resulting in extension of 3′ UTRs (22,49,67,68). In *ewg*, this suppression does not occur at the level of poly(A) site selection, and likely involves recruitment of later step cleavage factors, though the exact mechanism is not known (20). Here, we revealed that three U-rich motifs with regular spacing downstream of alternative poly(A) sites correlate with strong ELAV binding and site selection. We further revealed that poly(A) site binding affinity in the ELAV target *ewg* alters synapse formation, highlighting the physiological relevance of ELAV binding and its regulation of alternative poly(A) site selection. In fact, by fine-tuning EWG expression, ELAV has a critical role in determining the number of synapses through which motor neurons make contact with muscles (33,34). Likewise, the single ELAV orthologue in honeybees is required for learning and memory (14). Interestingly, it has been reported that the ELAV family member FNE can also rescue alternative poly(A) site selection in the absence of ELAV, and RBP9 has a role in determining synapse numbers (31,69). These findings indicate that ELAV family members share a common binding code and an important role in synaptogenesis. Moreover, regular spacing of U-rich sequences is conserved in ELAV/Hu family targets from *Drosophila* to humans, illustrating a general function of ELAV/Hu RNA-binding proteins that ELAV family binding specificity is both shared and highly conserved. In fact, transgenic *Drosophila* constructs with human HuR have been shown to regulate the same targets (31).

### Distinct *ewg*-like stem–loops are present in ELAV targets

Single-stranded pre-mRNA adopt higher order structures initiated by base-paired secondary structure (70). However, the relevance of RNA structure for binding of ELAV/Hu proteins has not been explored. Here, we identified an evolutionarily conserved stem–loop structure in the *ewg* ELAV binding site. Such *ewg*-like stem–loops are enriched in genes where ELAV regulates poly(A) site choice and are evolutionarily conserved. However, our footprinting assays are consistent with ELAV binding to single-stranded RNA. In fact, when U-rich motifs were completely base-paired ELAV could not bind. Possibly, RNA structure helps to expose U-rich motifs that are not base-paired in the *ewg* ELAV binding site, rather than providing a distinct feature that is recognized by its RRMs. Of note, the regular cleavage of RNase A in the footprinting experiments suggests a helical structure for the *ewg* stem–loop, which might facilitate presentation of U-rich sequences for binding by ELAVs. In any case, probing the functionality of RNA structure in an *in vivo* context is challenging (71), particularly with AU-rich sequences constituting a complex architecture for ELAV binding on an extended binding site.

### ELAV forms a defined complex, but can nucleate preferentially from 3′ to 5′

Various stoichiometric titration experiments revealed that ELAV can form a dodecameric complex on *ewg* target RNA (21). Through glycerol gradient centrifugation, we validated that ELAV forms a saturated complex that contains one RNA. For hnRNP A1, which forms a tetramer, it had been described that binding can extend by nucleation starting from a high-affinity binding site in HIV-1 *tat* exon 3 to block U2 snRNP binding and inhibit splicing (58–60). Intriguingly, ELAV also has a high-affinity binding section encompassing the proximal part of its extended ELAV binding site in *ewg* (21), and we show here that ELAV can nucleate, preferentially in a 3′ to 5′ direction. However, since ELAV binds 3′ of the poly(A) complex, this likely limits nucleation in a 3′ end processing context as suggested from previous cross-linking data (20).

For hnRNPs, a ‘beads on a string’ model similar to histones bound to DNA has been proposed based on EM visualizations (72). It is possible that nucleation occurs in the same mode, e.g. adding more ‘beads’. In fact, RNA is wrapped around the hnRNP tetramer as indicated from CLIP experiments in support of such a model (73), but hnRNPs have also been shown to form filaments and mediate neurotoxicity (74,75). In contrast, footprinting experiments with ELAV indicate that ELAV binding protects the RNA from nuclease access suggesting that a saturated ELAV complex has closed configuration with the RNA inside. Such a model is compatible with binding shorter RNAs, while hnRNP C RNA-binding sites are spaced apart by around 165 nucleotides (73).

Taken together, our analysis of ELAV binding properties and sequence requirements have brought about the advances necessary to start modelling ELAV binding at genomic scales feasible for identification of an underlying ELAV binding code. Of particular importance here is the identification of the critical spacing of U-rich motifs. Even though this spacing is mostly hidden in sequence degeneracy, our finding of distinct features like secondary structures which initiate complex formation and possible nucleation now need to be explored *in vivo* for a full understanding of ELAV/Hu function in regulating alternative mRNA processing.

## Supplementary Material

gkae826_Supplemental_Files

## Data Availability

All data generated or analysed during this study are included the supplementary information files.
