# Supplementary material for: Phylogenomic instructed target analysis reveals ELAV complex binding to multiple optimally spaced U-rich motifs
Source: Nucleic Acids Res. 2024 Sep 25;52(20):12712–26. doi: 10.1093/nar/gkae826 (PMC11551757; doi:10.1093/nar/gkae826)

Supplementary Data for

Phylogenomic instructed target analysis reveals ELAV complex binding to multiple optimally spaced U-rich motifs

DAVID W. J. MCQUARRIE[1,2] AND MATTHIAS SOLLER[1,2,3*]

Supplementary Results

Mutational analysis of the distal *ewg* ELAV binding site

Our footprinting analysis of ELAV bound to RNA Δ12 suggests that the right half of the stem loop structure (elements m3m and m3r) serves as an initial recognition site for ELAV. To confirm these data and assess the relative contribution of m3l, m3m and m3r AU_4 motif elements to high affinity ELAV complex formation, we designed deletions in Δ12 and tested them in EMSAs (Supplementary Fig. 1A and B). Since length also contributes to an increase in affinity for ELAV binding (1), we omitted vector sequence as well as the pyrimidine tract resulting in RNA M3H containing the entire m3 element (Supplementary Fig. 1A).

Although deleting either the m3l or m3r elements (Supplementary Fig. 1A, RNA M7H and M3P) resulted in a reduced binding affinity compared to parental M3H RNA, the reduction was more pronounced when m3r was deleted as in RNA M3P (Fig. 1A). Further subdivision of M7H to M7P and M9H each containing only one AU_4 motif almost abolished ELAV binding (Supplementary Fig. 1A and B, lanes 9-16). As ELAV complex formation could entirely be due to the length of the substrate RNA under these restrained conditions additional nucleotides derived from the vector were added to M9H resulting in M9S (Supplementary Fig. 1A). M9S does not bind ELAV demonstrating again that sequence composition is important (Supplementary Fig. 1A and B, lanes 17-20). We also note that the vector sequence and the

polypyrimidine tract absent in RNA M3H (Supplementary Fig. 1A) compared to RNA Δ12 decreased binding affinity about 20-fold.

The importance of AU_{4-6} motifs for sequence specificity has been shown in the context of the entire ELAV binding site from pA2 to exon I in UV-crosslinking and EMSA experiments (2). Since M7H comprises a minimal binding unit, mutations in AU_4 motifs and surrounding sequence were introduced to test for their functional significance in ELAV binding (Supplementary Fig. 1C and D). Surprisingly, substituting the two As with G residues in the AU_4 motifs of the m3m and m3r elements in M7H, or substituting an entire AU_4 motif of the m3m element with GC_4 resulted in a minimal effect (Supplementary Fig. 1C). Similarly, the effect of the $A(CU)_2$ mutation in the AU_4 motif of the m3m element was minimal (Supplementary Fig. 1C). In contrast, the $A(CU)_2$ mutation in the AU_4 motif of the m3r element reduced binding affinity 2.5-fold and $A(CU)_2$ mutations of AU_4 motifs in both m3m and m3r elements further reduced binding affinity, but binding was not completely abolished (Supplementary Fig. 1C and D, lanes 21-24).

As the two AU_4 motifs in RNA M7H are spaced by eight nucleotides they could bind two ELAV molecules suggesting that sequence surrounding the AU_4 motifs is also important for recognition by RRM1 and RRM2. Consecutive alignment of all the AU_{4-6} motifs from the *ewg* ELAV binding site revealed additional conservation of a PyU_2 motif 3' of the AU_{4-6} motif (Fig. 3A). To test if the PyU_2 motif is important for ELAV binding it was mutated to GA_2. Contrary to our expectations introducing the GA_2 mutation in either the m3m or m3r element showed a tendency to increase binding affinity for ELAV (Supplementary Fig. 1C and D, lanes 6-12). Only mutating PyU_2 motifs in both m3m and m3r elements resulted in decreased binding compared to the parental RNA (Supplementary Fig. 1C and D, lanes 13-16). A combination of the mutations in both AU_4 and PyU_2 motifs in the m3m and m3r elements resulted in a dramatic

decrease of ELAV binding (Supplementary Fig. 1C and D, lanes 17-20) suggesting that these two motifs together could form the sequence fitting into RRM1 and RRM2 of ELAV (Fig. 3A). In any case, these experiments indicate that we missed an essential aspect of ELAV complex formation and therefore focused on analysing spacing of multiple U-rich motifs as detailed in the main text (Fig. 2 and 3).

Supplementary Figures

Supplementary Fig. 1: ELAV complex formation is dependent on multiple U-rich binding sites

A and C) Schematic and sequence of ewg substrate RNA M3H and deletions (A), and M7H and point mutations (C) used for EMSA analysis. Averaged binding affinities from at least 3 experiments are shown on the right. Substrate RNAs used do not contain vector sequence. The few nucleotides added to allow in vitro transcription with an SP6 promotor are shown in small letters in 4A.

B and D) Representative gels of EMSA analysis using various uniformly ^{32}P labeled substrate RNAs (100 pM) as indicated on top. ELAV concentrations were 0.2 µM, 0.8 µM and 3.2 µM. Absence of ELAV is indicated on top of the first lane of each substrate RNA. Complexes were separated on 6% native polyacrylamide gels.

Supplementary Fig. 2: ELAV binding requires non-base-paired U-rich motifs

A) Schematic depiction of the synthetic RNAs used for EMSA analysis of based paired U-rich motifs on ELAV binding affinity. The *ewg* Δ12S control RNA was used as a high affinity positive control.

(B and C) Depiction of RNA secondary structure of 6xSM-AC (B) and 6xSM-A (C) Spacer sequences were either composed of A_6 or $[AC]_3$ repeats, altering their secondary structure by ensuring either full (C, red circles) or partial (B, blue circles) base pairing.

D) Quantification of ELAV complex formation on the two synthetic RNAs. The partially based-paired RNA substrate (6xSM-AC) formed the full ELAV complex while the paired RNA (6xSM-A) failed to. Kd values are shown in (A).

E) Example EMSA gels with either 100% A_6 spacers or with a ratio of 1:1 A and m^6A. ELAV forms an RNA-protein complex with the m^6A enriched substrate which is not visibly present for the A_6 spacer RNA.

Supplementary Fig. 3: RNA-seq of small RNAs pulled down from rELAV indicate no clear consensus motif and have no effect on ELAV-RNA complex formation in EMSAs

A) 5' end-labelled RNA pulldown from *E. coli* purified recombinant rELAV run on urea denaturing gels with and without the RNase A.

B) Validation of removal of small RNA pulldown by absorbance analysis (AU) of untreated and micrococcal nuclease (mcn) treated rELAV compared to a DNA control.

C) Purified rELAV untreated and treated with micrococcal nuclease run on a denaturing protein gel.

D) Read enrichment by genomic position in *E. coli BL21(DE3)*. The red dotted line indicates the cut-off used for comparative *de novo* motif analysis.

E) Sequence length enrichment of *E. coli* derived small RNAs pulled down from rELAV. Sequences above the cut-off (dotted grey line) were used for comparative *de novo* motif analysis.

F) Comparative *de novo* extended motif found from alignment of the sequences sorted based on D and E compared to the comparative *de novo* extended motif found from alignment of the

200-nucleotide poly(A) cleavage site downstream regions of ELAV regulated targets for comparison to the *de novo E. coli* motif. Red lines indicate aligning Us.

G) Example EMSA gels of ELAV untreated and treated with micrococcal nuclease with M7H and M9H RNA substrates. The *ewg*Δ12S control RNA was used as a high affinity positive control. Arrows indicate ELAV-RNA complexes.

Supplementary Fig. 4: Analysis of base pair conservation between *Drosophila* species

A and B) Structural position (A) and quantification (B) of complimentary base pair changes between closely related *Drosophila* species (*D. melanogaster*, *D. simulans*, *D. sechellia*, *D. yakuba*, and *D. erecta*) in the *ewg* ELAV binding site stem-loop secondary structure. Complementary base changes were classified as a nucleotide replacement required for stem-loop pairing maintained from *D. melanogaster* to another species. Base changes for each species are listed at their relative nucleotide positions.

Supplementary Fig. 5: Representative ELAV target stem-loops

A-D) Stem-loop secondary structures (A and C) and their free energy (ΔG) plots (B and D) from ELAV-regulated alternative poly(A) genes *5PtaseI* and *vn* in regions 200 nucleotides downstream of their annotated cleavage sites. ΔG plots were calculated at 37°C and relative energy at each point is shown.

Supplementary Fig. 6: Analysis of free energy in stem-loops

Average free energy (ΔG) for control and ELAV-regulated alternatively poly(A) genes (listed in Fig. 7B). 200-nucleotide regions upstream (labelled: 5' control; 5' ELAV ALEs) and downstream (labelled: 3' control; 3' ELAV ALEs) of poly(A) cleavage sites were analysed for

their stem-loop free energy values using mfold. Statistical significance was calculated from t tests (NS = not significant).

Supplementary References

1. Soller, M. and White, K. (2005) ELAV multimerizes on conserved AU4-6 motifs important for ewg splicing regulation. *Mol Cell Biol*, **25**, 7580-7591.

2. Soller, M. and White, K. (2003) ELAV inhibits 3'-end processing to promote neural splicing of ewg pre-mRNA. *Genes Dev*, **17**, 2526-2538.

Supplementary Figure 1

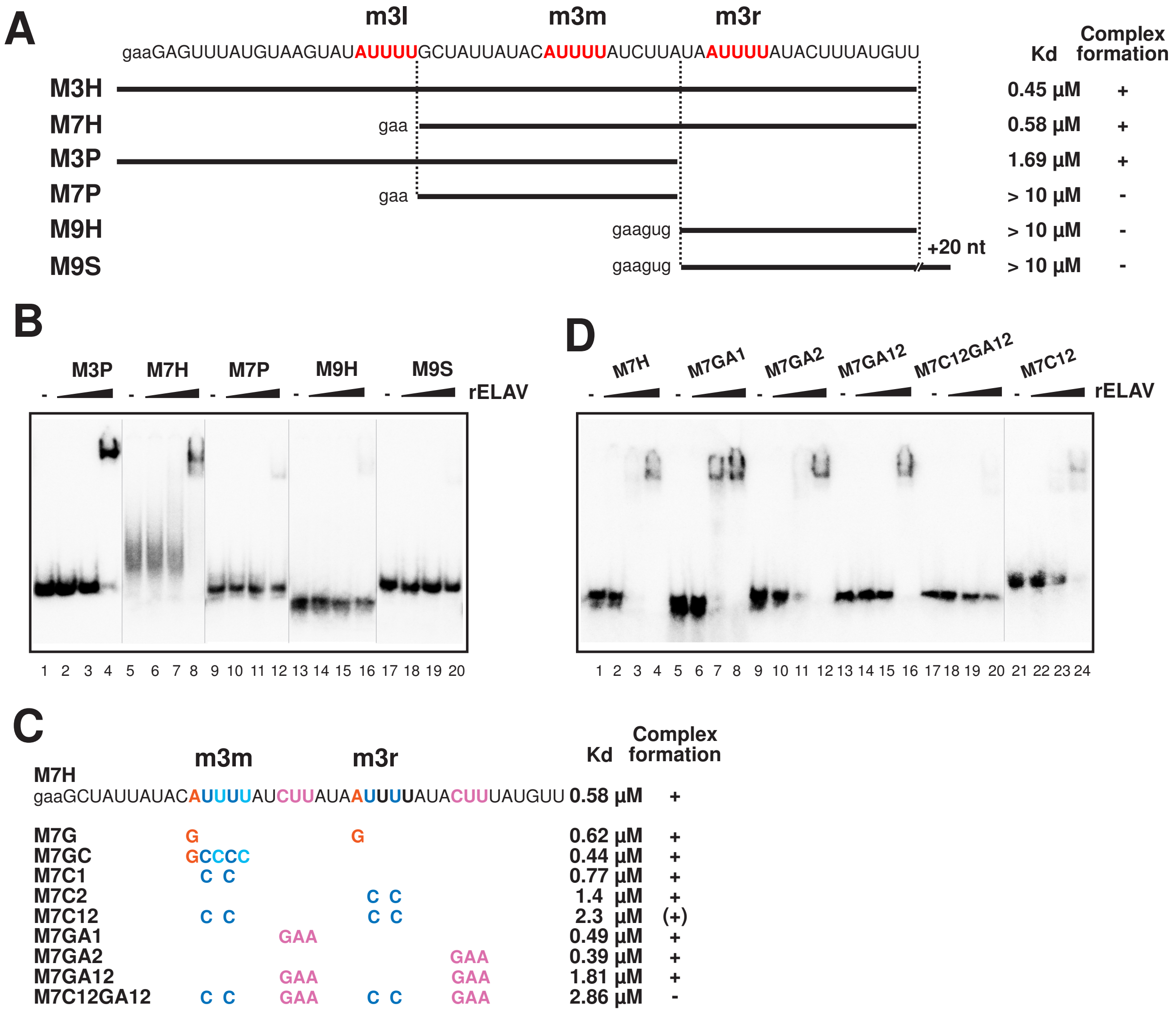

Supplementary Figure 2

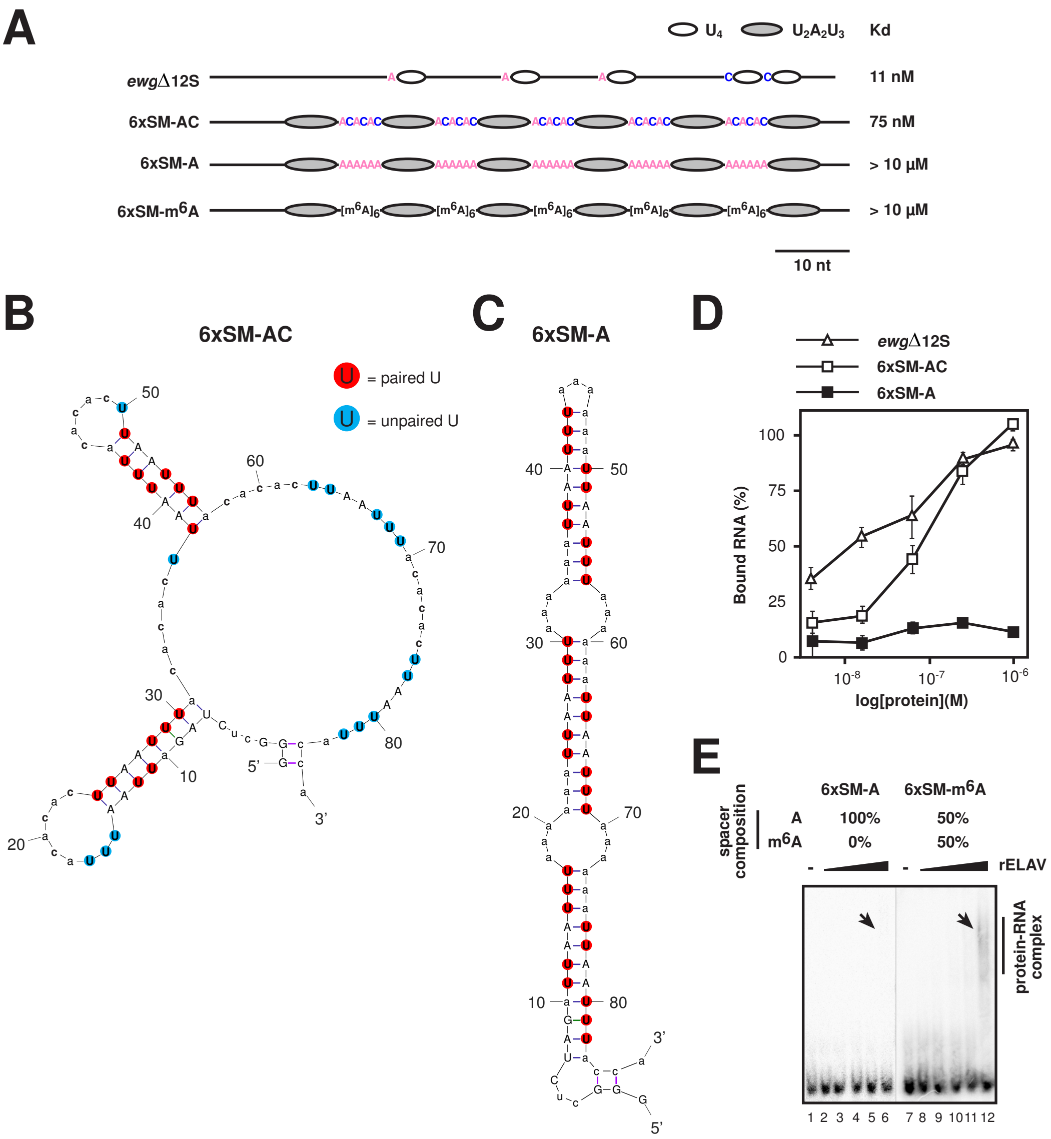

Supplementary Figure 3

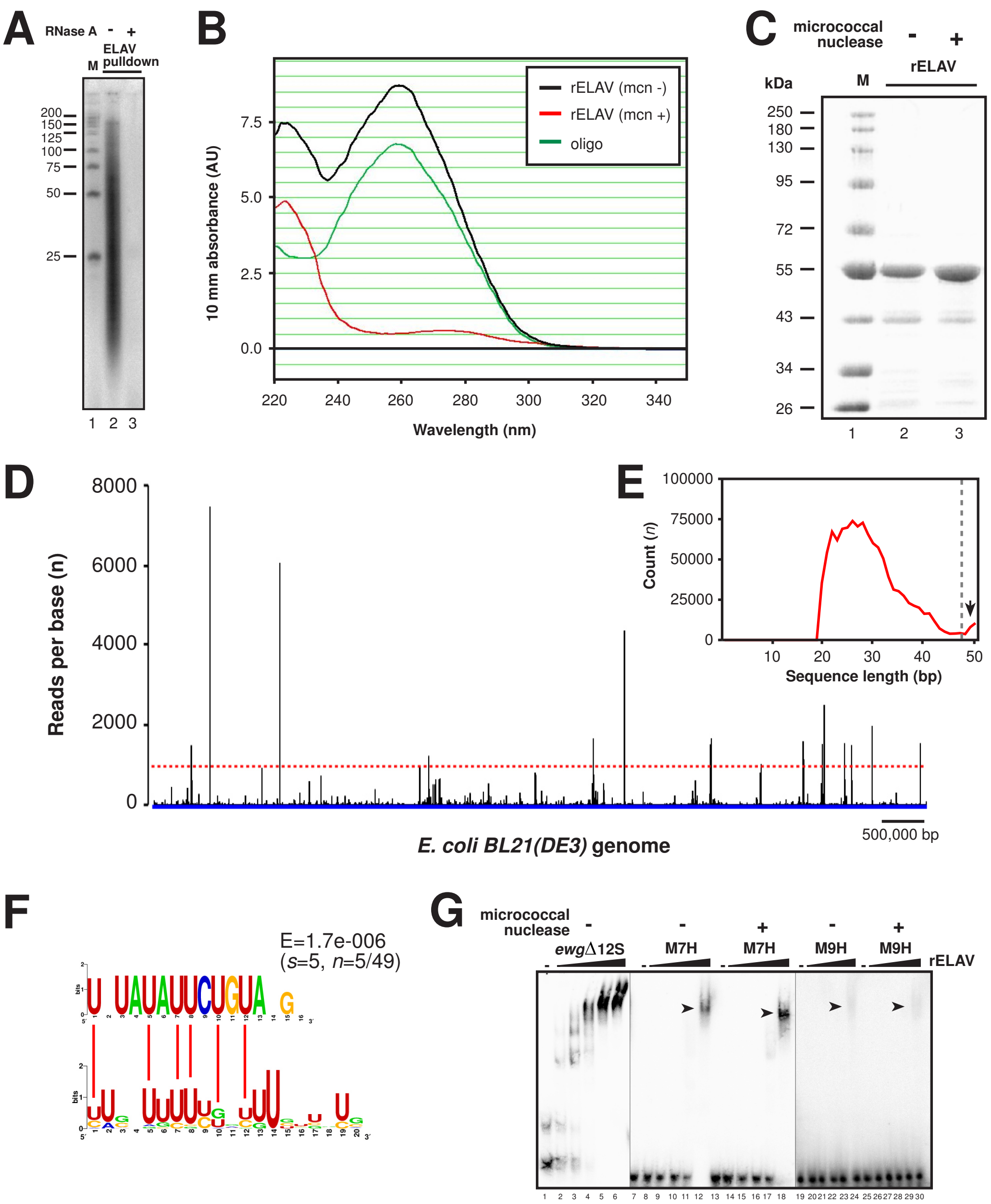

Supplementary Figure 4

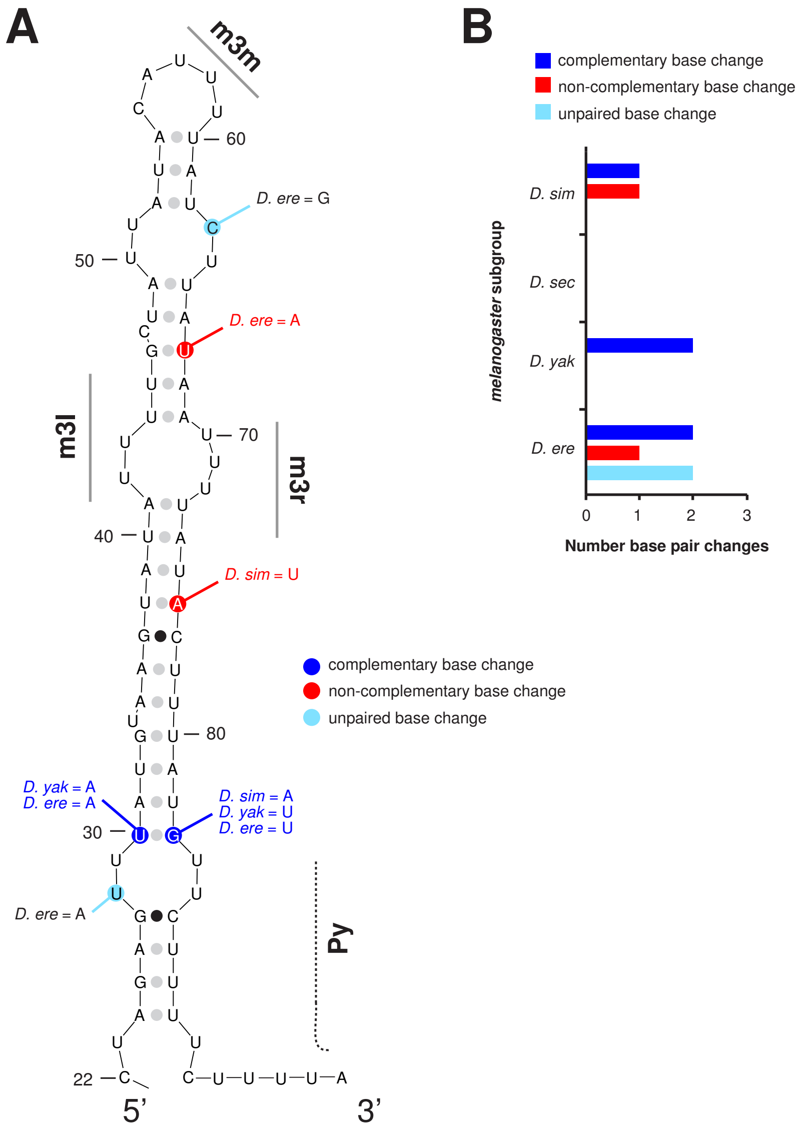

Supplementary Figure 5

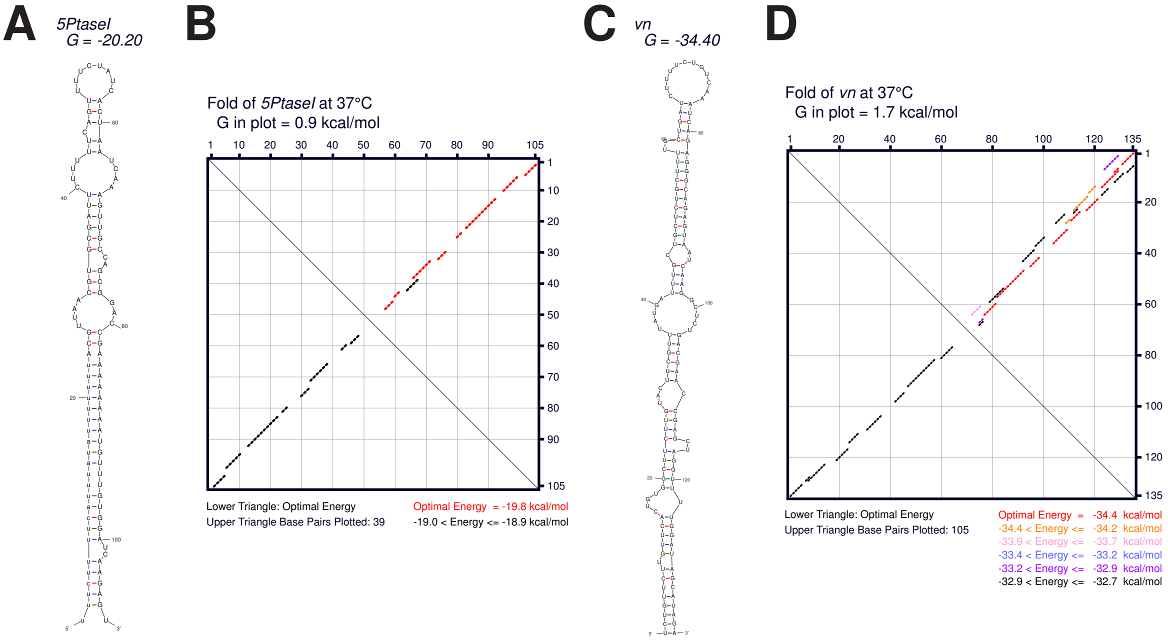

Supplementary Figure 6

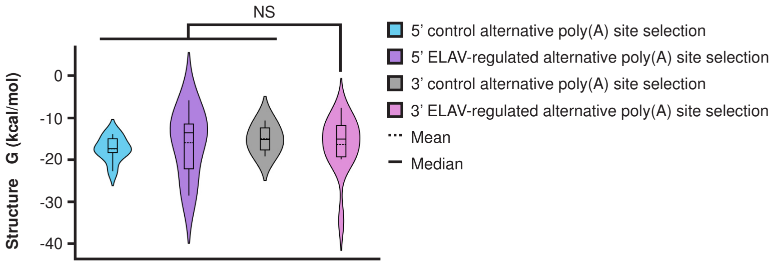

Supplement: gkae826_Supplemental_Files [file gkae826_supplemental_files.zip › McQuarrie et al. Supplementary Data rev2.pdf]
